# Transphobia as a social disease: discourses of vulnerabilities in trans men and transmasculine people

**DOI:** 10.1590/0034-7167-2022-0183

**Published:** 2023-11-13

**Authors:** Bernardo Haylan de Souza do Carmo Lobo, Gabriele da Silva Santos, Carle Porcino, Tilson Nunes Mota, Felipe Aliro Machuca-Contreras, Jeane Freitas de Oliveira, Evanilda Souza de Santana Carvalho, Anderson Reis de Sousa

**Affiliations:** I Universidade Federal da Bahia. Salvador, Bahia, Brazil; II Universidad Autónoma de Chile. Santiago, Chile; III Universidade Estadual de Feira de Santana. Feira de Santana, Bahia, Brazil

**Keywords:** Transsexualism, Sexual and Gender Minorities, Men’s Health, Health Vulnerability, Nursing Care, Transexualidad, Minorías Sexuales y de Género, Salud del Hombre, Vulnerabilidad en Salud, Atención de Enfermería, Transexualidade, Minorias Sexuais e de Gênero, Saúde do Homem, Vulnerabilidade em Saúde, Cuidados de Enfermagem

## Abstract

**Objective:**

to analyze the repercussions of transphobia on trans men’s and transmasculine people’s health.

**Method:**

a qualitative study carried out with 38 participants, 35 trans men and three trans men, who attended specialized transgender health services in Bahia, Brazil. In-depth interviews were carried out between June 2019 and February 2020. The Discourse of Collective Subject technique was used and interpretation based on the theoretical concept of transphobia.

**Results:**

transphobia has intra and interpersonal repercussions on the life and health of trans men and transmasculine people who attend health services. There were experiences of violence in the private space, fraying of family ties; discrimination in the school space; limitation in professional/work opportunities; barriers to self-care and access to health services; elaboration of trans identity protection strategies; consequences of transphobia on psycho-emotional health.

**Conclusion:**

transphobia is a social disease that affects different life and health dimensions. It causes damage to the socialization of trans men and transmasculine people, in addition to health service spaces as well as in family environments, schools, universities and at work, which result in non-adherence to self-care, distancing from health services and psycho-emotional distress.

## RESUMO

**Objetivo:**

analisar as repercussões da transfobia na saúde de homens trans e pessoas transmasculinas.

**Método:**

estudo qualitativo, realizado com 38 participantes, sendo 35 homens trans e três pessoas transmasculinas que frequentavam serviços de saúde especializado em trangeneridade na Bahia, Brasil. Realizaram-se entrevistas em profundidade entre os meses de junho de 2019 e fevereiro de 2020. Empregou-se a técnica do Discurso do Sujeito Coletivo e interpretação baseada no conceito teórico de transfobia.

**Resultados:**

a transfobia trouxe repercussões intra e interpessoais na vida e saúde de homens trans e de pessoas transmasculinas que frequentam serviços de saúde. Encontraram-se vivências de violências no espaço privado, esgarçamento de vínculos familiares; discriminação no espaço escolar; limitação nas oportunidades profissionais/trabalho; barreiras no autocuidado e acesso aos serviços de saúde; elaboração de estratégias de proteção da identidade trans; consequências da transfobia na saúde psicoemocional.

**Conclusão:**

a transfobia configurou-se doença social que repercute nas distintas dimensões da vida e saúde. Causa danos na socialização dos homens trans e das pessoas transmasculinas, além dos espaços dos serviços de saúde, como também em ambientes familiares, escolas, universidades e no trabalho, os quais resultam em não adesão ao autocuidado, distanciamento dos serviços de saúde e sofrimento psicoemocional.

## RESUMEN

**Objetivo:**

analizar las repercusiones de la transfobia en la salud de hombres trans y personas transmasculinas.

**Método:**

estudio cualitativo, realizado con 38 participantes, 35 hombres trans y tres personas trans masculinas que asistieron a servicios de salud especializados transgénero en Bahía, Brasil. Se realizaron entrevistas en profundidad entre junio de 2019 y febrero de 2020. Se utilizó la técnica del Discurso del Sujeto Colectivo y la interpretación basada en el concepto teórico de transfobia.

**Resultados:**

la transfobia tiene repercusiones intra e interpersonales en la vida y salud de los hombres trans y personas transmasculinas que acuden a los servicios de salud. Hubo experiencias de violencia en el espacio privado, deshilachamiento de lazos familiares; discriminación en el espacio escolar; limitación en las oportunidades profesionales/laborales; barreras para el autocuidado y acceso a los servicios de salud; elaboración de estrategias de protección de la identidad trans; consecuencias de la transfobia en la salud psicoemocional.

**Conclusión:**

la transfobia es una enfermedad social que afecta diferentes dimensiones de la vida y la salud. Provoca perjuicios en la socialización de los hombres trans y personas transmasculinas, además de los espacios de los servicios de salud, así como en los entornos familiares, escolares, universitarios y laborales, que se traducen en falta de adherencia al autocuidado, alejamiento de los servicios de salud y sufrimiento psicoemocional.

## INTRODUCTION

During the development of society and science, the concept of health and disease was constantly changing and changing. Transgenderism, until recently, was considered a psychopathological condition. In 2018, the World Health Organization (WHO), in the 11^th^ review of the International Classification of Diseases (ICD), removed the classification of “transsexualism”, which considered transsexuality as a mental illness^(1)^. With the change, the term came to be called “gender incongruity”, inserted in the chapter on sexual health^(2)^. Due to this historical pathologization of transsexuality(ies), transsexual and transvestite men and women remain susceptible to the negative impacts resulting from the non-recognition and legitimation of their identities by society, potentially generating psychological distress, illness, violation of rights, exclusion and discrimination.

Within the scope of gender identity demarcation, cisgenderity is based on the understanding that biological sex, represented by the external genitalia’s anatomical conformation, which is consistent with gender identity, is an understanding that needs to be updated with regard to changing the notion that this is a single and linear identity. In another light, from a perspective of subversion and transgression of the cis and heteronormative system, transgenderism is understood as the non-recognition belonging to the gender that was determined at birth and its determined social expressions^(3)^. The imposition and structuring of heteronormative cisgenderism still prevails in society, which regulates the agreement between sex at birth, gender identity and sexual orientation, mischaracterizing people who do not recognize themselves as such. Such conceptions and understandings subject transgender people daily to a continuous stressful process of transphobia and psychic illness^(3)^.

Thus, transphobia has had a deleterious impact on the life and health conditions of transgender people around the world, which is a phenomenon typified by discrimination, aggression and rejection of the way trans people construct their gender identities^(4)^. Especially in Brazil, transphobia has been responsible for the violent death of thousands of people, due to hatred attributed to the gender dimension. According to the Brazilian National Association of Transvestites and Transsexuals (ANTRA - *Associação Nacional de Travestis e Transexuais*), Brazil is the country that most kills transvestites and transsexuals in the world, and ranks among countries in terms of safety for the LGBT population. Therefore, the life expectancy of this population in Brazil is 35 years, less than half of the estimated national population average of 75 years^(5)^, although studies in this field are still limited in the Latin American context, which reveals a gap in the scientific knowledge on the subject.

Violence is not limited to physical expressions, but can be perpetuated in everyday life, advocating it and exposing social vulnerabilities and health, in all its dimensions, crossed by stigma, isolation, loneliness and erasure of trans existence and visibility^(6)^. For these reasons, it is necessary that strategic actions for coping with transphobia may imply improvements in training, in clinical-assistance practice, in research and in care/health service management, with a view to redirecting work in nursing and in other areas of health in the care of trans men and transmasculine people, actions among which include promoting access to an assisted, safe, welcoming and unique gender transition^(^7-8^)^. Therefore, in line with the need to implement the aforementioned actions, this study reveals direct contributions to nursing science and practice. Therefore, this study was guided by the guiding question: how does transphobia affect the health of trans men and transmasculine people?

## OBJECTIVE

To analyze the repercussions of transphobia on trans men’s and transmasculine people’s health.

## METHODS

### Ethical aspects

This study complied with ethical recommendations in all its phases. To guarantee anonymity, the participants were identified collectively through the methodological figure called DSC.

### Theoretical-methodological framework

The results were interpreted in the light of the theoretical-political and socio-anthropological perspective of transgenderities in health, more specifically with a focus on transphobia, structured in the concept analysis proposed by Podestà^(9)^, dedicated to designating and analyzing the multiple forms of violence committed against transgender people. Its reach potential can affect transvestites, non-binary people and people with other gender identities, due to the gender norm imposed by cisgenderism^(9)^.

### Study design and setting

This is a qualitative study^(10, 11)^. The research was carried out in two municipalities in the state of Bahia, Brazil, and followed the Standards for Quality Improvement Reporting Excellence (SQUIRE 2.0) criteria for research protocol construction.

### Data source

Participants were 35 people with self-reported gender identity as trans men and three with transmasculine gender identity. In this study, a trans man was considered a person whose gender identity or expression (male) is different from his/her sex (female) assigned at birth and a transmasculine person; person whose identity has some relationship with being a man or with masculinities. In this regard, it is noteworthy that, in the definition of trans man and transmasculine person adopted by the researchers, there is no reference to biological sex. Such definitions were drawn up anchored in the scientific literature on the subject^(12)^.

The research team was composed of a trans woman, a cisgender woman, a trans man, a non-binary person and two cisgender men, with undergraduate training in nursing and psychology, specialization in the form of residency, master’s and doctoral degrees in nursing and health. Two researchers responsible for the study already had contact with part of the investigated group (first sample group), through teaching and research activities in partnership with the university and groups with guidelines aimed at Lesbians, Gays, Bisexuals, Transvestites, Transsexuals/Transgenders, Queers, Intersex, Asexual, Pansexual, Non-Binary/Binary and other identities (represented by the + identification) (LGBTQIAPN+).

Participants were accessed in diverse spaces of belonging, such as nightclubs, bars, meeting points, universities, meetings of collectives and social movements, among others, and in partnership with the Municipal Department of Social Development, in the LGBTQIAPN+ Population Attention Division sector, and in an outpatient clinic specialized in caring for trans people, both in the state of Bahia, Brazil. Initially, they were accessed through the consecutive recruitment strategy called snowball^(13)^, which enabled the creation of a first sample group called “seeds” (ten participants). This first group was encouraged to indicate new participants, who made up the second sample group (28 participants), which was called “children of the seeds”.

The technique used in participant selection made it possible to reach the theoretical sampling^(14)^, based on collected data theoretical density and data theoretical saturation determination^(15)^, which considered the criteria of data co-occurrence, convergence and complementarity. People with gender identity, transvestites and those residing in other states were excluded from the study. Among the 42 invitations made, four people refused to participate in the study, with the justification of lack of time to carry out the interview.

### Data collection and organization

Data production took place between June 2019 and February 2020, through individual in-depth interviews^(16)^, scheduled according to participants’ availability, carried out in a single meeting, guided by a semi-structured script composed of questions: could you talk about you? Could you tell us about your gender/transgender transition? Could you describe how your health care takes place? Could you tell us about your experiences in health services? Individual interviews were carried out in the academic space and in the facilities of social and health care services, in a reserved place, with guarantee of individuality, privacy, preservation of image and anonymity of the information collected. They had an average time of one hour, and were recorded, transcribed in full, coded and organized with participants’ authorization. After the transcripts, the interviews were made available to participants, who assessed and agreed to the coding and analysis stage. The recommendations of the criteria established by the Consolidated Criteria for Reporting Qualitative research (COREQ) were followed.

### Data analysis

The data obtained were coded and categorized under the support of NVIVO®11, and, after validation by the research team, were submitted to Discourse of Collective Subject (DSC) analysis. This technique allows revealing methodological figures from the discourses, such as key expressions (KE) and central ideas (CI), which composed the discourse synthesis (DS), which made it possible to carry out discourse convergence and complementarity from line-by-line reading and the search for occurrences, in addition to gathering the data that expressed the phenomenon of thought and collective representation^(17)^. The categorization followed a second stage of validation by the research team.

The categories were organized and interpreted based on elements proposed by sociopolitical and anthropological theories that conceptualize and explain the transphobia phenomenon^(9)^, namely: specific violence, abjections; naturalizing and normalizing social sanctions; cisgender-dependent gender normalization; punishment and degradation of social identity; and genocide.

## RESULTS

The results are presented based on the participants’ characterization data (Chart 1), an explanatory model of the investigated phenomenon and the synthesis speeches, expressed through Central Ideas.

**Chart 1:** Study participant characteristics - trans men and transmasculine people, Salvador, Bahia, Brazil, 2022

**Personal, sociodemographic and economic characteristics**	**Age:** 18 to 30 years; **Self-reported race/color:** brown; **Average family wage income:** less than one minimum wage; **Religion/Belief:** Catholic; **Schooling:** incomplete elementary school; **Employment status:** employment relationship without a formal contract; **Sexual orientation:** heterosexual.
**Health status/condition**	**Health status:** regular; **Presence of the Brazilian Health System (SUS – *Sistema Único de Saúde*) card:** used; **Social name use on the SUS card:** not used (at the time of the survey); **Health system use:** public service in greater prevalence; **Access to the system for performing masculinizing mammoplasty surgeries:** private health service; **Vaccination status:** regular; **Chronic diseases:** none; **Continuous medication use:** testosterone, without medical prescription; history of sexually transmitted infection: syphilis and viral hepatitis.
**Social vulnerabilities experienced in health services**	**Religious intolerance:** experienced; **Racism:** experienced; **Transphobia:** experienced; **Violence:** experienced – verbal expression.

### Participant characterization

**Figure 1:**
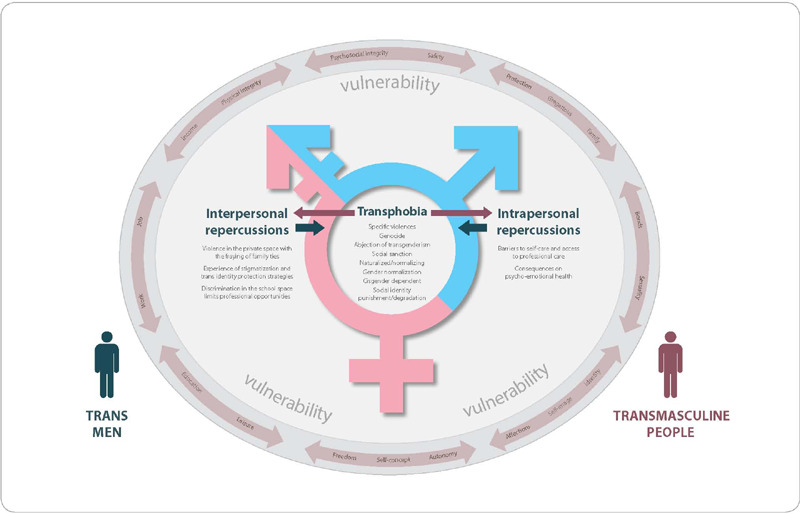
Explanatory model of the psychosocial repercussions caused by transphobia for trans men and transmasculine people, Salvador, Bahia, Brazil, 2022

It is noteworthy that, although the study focused on the scope of health services, the speeches covered other dimensions of transgender life and sociability, such as family, school, university and work environment. Such a scenario is represented in Figure 1 below. In this figure, the cyclical way in which the constituent phenomena of transphobia operate and the repercussions of an interpersonal and intrapersonal nature caused to trans men’s and transmasculine people’s life and health, in a context permeated by multiple vulnerabilities.

### Discourse synthesis 01: Interpersonal repercussions

The interpersonal repercussions perceived by trans men and transmasculine people identified in participants’ discourse were diverse and are organized in this category to reveal their breadth and complexity.

### Central idea 1A: Violence in the private space with weakening of family ties

In this CI, participants reported on the conflicts experienced in the private space, attributed to family members’ refusal to accept transgenderism, which are based on religious beliefs, on the notions of sin and on the pressures exerted by them in an attempt to adjust participants’ bodies to the heteronorm. These conflicts are marked by physical abuse, abandonment with expulsion of the trans person from the family environment, ruptures in coexistence and weakening of family ties, represented by Chart 2.

**Chart 2:** Discourse of Collective Subject of violence in the private space with weakening family ties experienced by trans men and transmasculine people, Salvador, Bahia, Brazil, 2022

Key-Expressions	Discourse of Collective Subject
Non-acceptance Fights Rejection Sin Fear Revolt Alcohol and other drug use Distancing Break of bonds	[...] *the relationship has always been very difficult. My parents did not accept me and the conflicts due to being transgender were constant, making the situation unsustainable. The justifications for fights and rejection were strongly influenced by religion, as my parents saw transgenderism as something sinful and wrong. I even got beaten up by my father, and because of that, I started to feel fear and revolt and it was during this same period that I started drinking and also using drugs. When they found out about my position on transgenderism, they threw me out of the house. Due to the transphobia that also affected the environment in which I lived, I ended up moving away from the whole family and with that the affective bond was affected*. (DCS)

### Central idea 1B: Experience of stigmatized people in the development of trans identity protection strategies

This CI explained that trans men and transmasculine people, when perceiving the pressures to frame their identity in a rigid norm, and when experiencing violence both in private and public spaces, they perceive the threats and seek to protect both their physical and emotional integrity by adopting the self-isolation, limiting social contacts, acting with the concealment of body marks that differ from their gender identity (Chart 3).

**Chart 3:** Discourse of Collective Subject of discrimination in the school space that limits professional opportunities experienced by trans men and transmasculine people, Salvador Bahia Brazil, 2022

Key-Expressions	Discourse of Collective Subject
Isolation Fear Prejudice Illness Violence Discrimination Embarrassment in public Disrespect Denial Family alienation	[...] *I lived and still live largely isolated from people. I live discreetly in my neighborhood, as I am afraid of the violence generated by transphobia. Most of the time, I hide, because I’m afraid of being noticed as a feminine trait in me, like my breasts, and that being the target of violence. I avoid getting home late and hanging out on the street, and I spend most of my time alone. Going out into the street is always sickening, because I already know that I will face a lot of prejudice and violence. I have been through many embarrassing and discriminatory situations in public spaces, such as banks, health services, legal services and companies, making me feel embarrassed about my transgender identity, which is often not respected, such as the denial of the social name. I don’t have many friends, not much contact with family. What gives me great support is the group of trans friends and trans people allied with social movements and LGBT groups. It is also very difficult to get along at work, especially in places where transgenderism is not well accepted. Having to hide my breasts so as not to notice the volume and delegitimize my passing as a man is very uncomfortable and distressing, because I have to spend all the time thinking about this possibility*. (DSC)

### Central idea 1C: Discrimination in the school space limits professional opportunities

In this CI, the trans men and transmasculine people investigated recalled the experiences of discrimination suffered since childhood in school spaces and how much these experiences had a negative impact on their emotions, produced a feeling of shame and discouraged them from studying, which contributed to the low educational and training performance and the evasion of school and academic spaces to pursue a professional career in adult life (Chart 4).

**Chart 4:** Discourse of Collective Subject of discrimination in the school space that limits professional opportunities experienced by trans men and transmasculine people, Salvador, Bahia, Brazil, 2022

Key-Expressions	Discourse of Collective Subject
Discrimination in the school environment	
Problems at school Lack of welcoming Discomfort Pain Violence Masculine woman Yield decrease Withdrawal from studies Job difficulty Daily prejudices Disrespecting the name	[...] *since childhood, I had problems with the school, which never welcomed me. School has always been a place of great discomfort and pain, as it was a place of much violence. Several times, I failed to take advantage of recess time and stay inside the classroom to avoid violence. Since that time, I was already seen as a boy, but I didn’t have that understanding yet, and, because of that, I was seen as a masculine woman, and that was also the case in adolescence. Because of these problems, I didn’t have good income and I gave up studying. I was ashamed, afraid and all this prevented me from learning, from concentrating and from facing the challenges that school presented. The reflection of all this I try to recover nowadays, because without schooling everything became more difficult, including finding a job. I even get some opportunities, but they all require training. Getting to university is another challenge that seems unattainable, because I know that I will have to face more difficulties, including the breaking of daily prejudices that I suffer, such as, for example, respect for my social name. I had to fight to have my name on the attendance lists, in the registration system, and even so I suffered transphobia from professors*. (DSC)
Professional limitations in the job market	
Financial difficulty Difficulty in formal employability Hiding place Delegitimization of trans passing Discomfort Anguish Loss of food quality and access to health services – follow-up, hormone therapy, surgeries, survival Vulnerabilities	[...] *all of this affects the quality of my diet, access to going to the health service to carry out my follow-up, hormone therapy, as the health system does not offer hormones for free, nor achieving breast adjustment surgery and even survive. Because of this, I have noticed many women and also trans men vulnerable to prostitution and trafficking*. (DCS)

### Discourse synthesis 02: Intrapersonal repercussions

Among the intrapersonal repercussions caused by transphobia in the lives of trans men and transmasculine people, physical and mental health problems were highlighted, which compromised self-care and access to the health system.

### Central idea 2A: Transphobia barriers to self-care and access to professional care

This CI showed that the transphobia experienced in health institutions has an impact on participants’ motivation for self-care, encouraging them to adopt unsafe measures to access hormone-regulating drugs. Furthermore, the absence of services that consider the unique needs of the trans population reduced the level of confidence of trans men and transmasculine people investigated. This prevented them from creating bonds between users and health workers, which implied low adherence to health promotion care and negligence of professional attention to care during the reproductive cycles, in the prevention of cancers related to the reproductive system and to the care related to hormones (Chart 5).

**Chart 5:** Discourse of Collective Subject of transphobic barriers to self-care and access to professional care experienced by trans men and transmasculine people. Salvador, Bahia, Brazil, 2022

Key-Expressions	Discourse of Collective Subject
Daily transphobia Contempt with care Difficulties in services Institutional violence Adoption of risky practices Hormonization on its own Difficulty accessing hormones in the SUS Challenges Discomfort and fears Transphobic professional approaches Risks to physical, sexual and reproductive health Loneliness Search for other sources of care: groups of trans men	[...] *due to the transphobia experienced daily, I started to despise taking care of my physical health. This fact is mainly due to the difficulties I have already faced in health services, which made me stop attending, to believe in the work of professionals, because I have already experienced a lot of institutional violence. Due to the need to carry out changes in my body, I ended up performing some risky and dangerous practices, such as using hormones on my own, and this is due to the fact that I had already suffered judgments from health professionals when asked to administer the hormones that are prescribed by the doctor of the municipal health network. And another complicating factor is that the SUS does not offer hormones for free and, with the financial difficulties, I end up not having access. Having to carry out gynecological care has also been a challenge that I struggled with for a long time, mainly because I did not feel comfortable with professionals’ approach and because of fear of transphobia. Many trans men do not undergo breast and uterus examinations due to this fear. I do not feel that health professionals are prepared to deal with physical health issues of trans men, such as, for example, menstruation, use of hormones and their risks to physical health, dietary care, sexual and reproductive practices, pregnancy and childbirth as well as the transition process, which ends up being lonely, most of the time without adequate follow-up, which makes me look for other sources of access to care, such as the groups of trans men present on social networks available on the internet and in collectives and support groups* [...] *all of this affects the quality of my diet, access to going to the health service to carry out my follow-up, hormone therapy, as the health system does not offer hormones for free, nor achieving breast adjustment surgery and even to survive*. (DSC)

### Central idea 2B: Consequences of transphobia on psycho-emotional health

In this CI, participants presented the repercussions of transphobia on their psycho-emotional health. They pointed out the frequent outbreak of feelings of inadequacy regarding body and self-image, self-depreciation and suicidal ideation, and highlighted that there is denial of the name with which they identify in public spaces. As a result, they alienate them, including services that should take care of mental health. Additionally, they assessed the experience as the most vexatious and causing discomfort ever experienced (Chart 6).

**Chart 6:** Discourse of Collective Subject of the consequences of transphobia on the psycho-emotional health experienced by trans men and transmasculine people, Salvador, Bahia, Brazil, 2022

Key-Expressions	Discourse of Collective Subject
Decision conflict Sexuality and body perception impairment Negligence Contempt Disgust Strangeness Mental health impairment Anxiety Agitation Irritability Body dysphoria Suicide Psychiatric care CAPS Lack of welcoming Disrespect for trans identity Sadness Revolt treatment abandonment	[...] *the most difficult were not the moments of conflict of decision with my gender identity, but the transphobia that I started to experience, affecting not only my sexuality, my perception of my body, my image and personality, but my existence. It has been almost a daily battle to survive in the face of transphobia that demonizes, exoticizes, neglects, harasses and denies the trans experience. They are games in bad taste, looks of contempt, disgust, strangeness, lack of acceptance and sensitivity that even happens in health services. These experiences generate real compromises in my mental health. I feel constantly anxious, agitated, irritable, with high stress, change in mood, appetite, in addition to the body dysphoria I’ve already experienced. I even tried to commit suicide, with the desire to end my life, due to a severe depression that I experienced. Due to the serious mental health situation I was in, I sought psychiatric care at a CAPS, but even in that space I already suffered transphobia and did not receive the care I needed. Several times, I was called by my registered name, delegitimizing my male identity. That situation humiliated me, made me sad and angry, and became the reason why I abandoned, missed subsequent appointments, abandoned treatment and became resistant to seeking a health service*. (DCS)

## DISCUSSION

This study investigated the collective discourse of trans men and transmasculine people, and showed that transphobia causes inter and intrapersonal repercussions that affect physically, socially and psychologically life and health in different ways. Transphobia becomes demarcated by the manifestation of social isolation, stigma, prejudice and discrimination as multiple phenomena that can influence concomitantly. These phenomena can result in actions that formulate abasement (by formulating a condition of expression of baseness, contempt and degradation of identity) to transgenderism^(9)^, and contribute to the emergence of barriers in accessing health services as well as other spaces of socialization, often indispensable to everyday life, as in educational and professional training environments in environments and/or work, public and/or private offices, leisure environments as well as public spaces^(3, 7, 8)^.

The findings of this study also indicated that transphobia had repercussions on the physical and mental health of those investigated, motivated by the denial of essential human rights, such as the free, safe and healthy exercise of citizenship and the expression of their identities as well as by institutional violence and degradation of family ties, marked by the distancing from the family environment and housing/shelter. This set of complex issues presented can configure different aspects of vulnerability to which trans men and transmasculine people are exposed. As a result of transphobia, they indicate the necessary expansion and strengthening of care in nursing and other areas of health to this population’s demands and needs.

The occurrence of specific violence^(9)^ triggered by transphobic practice causes family life unsustainability, increasing social/health vulnerabilities, through the initiation of the abusive alcohol and other drug use during the transition to the self-reported gender^(18)^. Family spaces configured by fragile, stressful and violent dialogical relationships can influence the departure of transgender people from the nuclear spaces of the family and the experience of patrimonial and moral damages^(19)^. Furthermore, family non-acceptance can result in the suicide of trans men and transmasculine people, which appears at high levels when compared to cisgender men^(^18,20^)^. Such a scenario should reinforce the work in family nursing, with advances in questioning transgenders in family/reproductive planning actions, review of the concept of family, redefining the use of instruments/public devices and health programs aimed at adolescents and their families, with a view to improving cohesion, harmony and family ties in the context of transgenderism^(21, 22, 23)^.

According to a survey carried out with trans men from the five regions of Brazil, 80.7% of participants claimed that their own home was the most disrespectful environment they attended^(24)^. With contexts demarcated by religion, whose logic of sinfulness, the idea of a “dirty, unworthy, sinful and erroneous body”, can intensify the transphobia perpetrated by family members against the “gender-divergent person”. This reality can revert to triggering negative feelings/emotions, reflecting the indignation and impact suffered by those who should be the primary source of support/ support, safety/protection and expression of affection/love^(23, 24)^.

Therefore, action in clinical nursing practice domains were: safety/protection; stress control; roles and relationships. The latter was aimed both at trans men and transmasculine people and at their family members (performance of social roles, social interaction, socio-affective relationships, tension in the role of caregiver, conflict in the role of father/mother, family relationships - dysfunctional family processes, bonds harmed)^(25)^. Thus, they need to be strengthened in nursing therapy with families of trans people, in interface with other areas of health and social/ human sciences, such as psychology, pedagogy, occupational therapy, social work.

The coexistence of trans and transmasculine men in society has been hostile and perverse, sometimes lonely, due to the stigma and discrimination that social isolation imposes^(4, 18)^, due to the naturalized/normalizing social sanction against transgenderism^(9)^. The quest to cope with gender stereotypy and scarce social support has been experienced on a large scale by the trans community, which still lacks public devices that guarantee its safety and social and human protection.

Faced with this difficult circumstance, the fear of suffering violence in the spaces where they transit often emerges, which generates greater restriction to domestic environments (for those who are not homeless), and the decrease in socio-affective interaction, free right to come and go/participate/live in society^(20)^. Therefore, it is recommended that nursing professionals and professionals from other health areas act in the face of ineffective coping, the feeling of impotence and impaired resilience, which may derive from the consequent stigmatization experienced by trans men, in dialogue with public authorities, public policy makers, educational health managers, activists, community leaders and the organized social movement.

Faced with these circumstances, trans and transmasculine men can become discreet, almost invisible to people’s eyes, due to the fear of expressing their identities socially and establishing their body readings in front of others, through the fear of delegitimization, embarrassment, humiliation and other forms of violence and social oppression^(23, 24)^. Under this issue, our findings in this present study revealed that this fact may be related to the way trans men and transmasculine people deal with self-image and body image, as in the case of the breasts, an organ that compromises the body archetype “passing” and configuration and the male figure aesthetics^(23)^ in social imaginary. In view of this, it is salutary to create specific lines of care for specialized, comprehensive and equitable health care in the health network available to the trans population, in addition to qualification of nursing teams in health care promotion with a view to valuing trans identities, self-image/perception and self-concept.

The problem of transgender self-image, which can be deteriorated by transphobia, can be enhanced by public services’ weaknesses in offering actions that contribute for promoting trans visibility, namely: support groups; formation of collectives; construction of affective networks with a view to friendship between the trans population that uses the services (“belonging” groups) and the interaction (“among peers”) among workers, health professionals and users^(26)^. In this way, nursing teams can be useful in building and monitoring these relationships, in addition to encouraging trans men and transmasculine people to raise their self-esteem and build/maintain self-care, with a view to establishing healthy lifestyle habits.

Contributions derived from social support can also open up an opportune space for the increase of nursing interventions aimed at producing technologies for the trans population^(27)^, such as the establishment of nursing consultation scripts and the creation of care plans, through the formulation of nursing diagnoses, outcomes and interventions focused on gender/transgender identity.

What has been described, up to this point, as social exclusion, body heteronormativity of bodies, rejection, murder and countless other physical, mental, symbolic or structural violence, makes up the genocide of trans people^(8, 18)^. Given this context, of the 984 cases of murders perpetrated against transgender people between 2002 and 2016 in Brazil, revealed in a survey, 76.8% were carried out on public roads, which puts this portion of the population in a state of perennial vulnerability^(6)^, also pointed out in another investigation carried out in the Northeast region^(24)^.

The constant struggle between the invisibilization of trans identities and the rejection of transgender persons, also reinforced by our findings, draws attention to the union of interprofessional, transdisciplinary and intersectoral efforts regarding violence coping, which is a result of the hegemonic and compulsory patriarchal cisgenderism. This has led to the emergence of psychiatric pathologies with persistent clinical manifestations, difficult-to-manage psycho-emotional somatizations, in contexts of self-mutilation and suicidality, in addition to homicides, which have claimed these people’s lives^(3)^.

It follows that there is, within the scope of the manifestation of transphobia, a fine line between what violence is perceived and faced in health services and in other institutions. This problem can contribute to the naturalization of violence (transphobia), even if it can be configured as self-injurious and/or self-defeating for the trans population. Thus, the transphobic psychosocial impact perpetrated in public and/or private departments (financial institutions, legal sectors, work organizations) can confirm the denial and delegitimization of transgender people in these spaces, facilitate/reinforce the reaffirmation of institutionalized transphobia, in the face of repetition, and violate already acquired legal constitutional principles, such as social name use, name and gender rectification^(4, 28)^. Thus, the expansion of the psychosocial care network and the work in mental health nursing directed at trans men and transmasculine people become urgent.

The existence of transphobia in health services constitutes an error and serious violation of the guarantee of the SUS principles, which provides for universality, comprehensiveness and equity in health care^(23)^. Although there is, on a daily basis, non-compliance with these principles and assumptions contained in the Charter of Rights of SUS Users (*Carta dos Direitos dos Usuários do SUS*)^(23)^ (a disturbing problem for the health of transgender people), transphobia needs to be seen as a significant public health problem. Deleterious effects of transphobia imply a decrease in survival, precariousness in health care production, distancing of the trans population from health services and distancing from healthy community spaces for coexistence (parks, squares, outdoor gyms). Moreover, impairment of child growth/development, damage to learning, intellectual deficits are evidenced, through cisheteronormativity^(28)^.

Transphobia can cause difficulty in the insertion of trans men and transmasculine people in the formal job market, in maintaining employability, income and financial security^(29)^, impacting the livelihood of trans people^(28, 29, 30)^, given the institutional regulation of cisgender dependent gender^(9)^. Thus, financial precariousness brings real harm to physical health: low quality of life; difficult access to inputs necessary for desired body changes (acquisition of safe and quality hormones); non-compliance with therapeutic agendas (clinical consultations/assessments, follow-up, diagnostic tests, surgical or non-surgical body procedures)^(23)^. In view of this, the need to maintain income may imply the adoption of strategies that guarantee transmasculine “passing”, by hiding any characteristics socially perceived as feminine, which may explain the constant use of a device called “binder”, used by many trans men and transmasculine people for devices to disguise the breasts, compressing them, considering the fear of punishment and degradation of identity in the work environment^(9)^, as evidenced in this study.

The abandonment of transgender people from school environments requires broad attention from the health sector. There is evidence in the literature that points to the school institution as a promoter of transphobia, given the occurrence of rejection, sanding and bullying, which implies school dropout and evasion, both for trans people and transvestites^(30, 31)^. Thus, when schools do not have pedagogical strategies to deal with transgenderism in these spaces, the installation of a transphobic culture is derived, as evidenced in the present study. In this regard, transphobic contexts may also be perpetuated in university environments, which contributes to delegitimizing the presence and permanence of this population in universities.

Derive from this problem described above the perennial need to advocate for minimal rights, the management of conflicting relationships between classmates, professors and employees and the management of psychological distress^(30)^. Therefore, it is essential for nursing professionals to articulate with the devices available in the network (schoolchildren, universities, training centers), considering, mainly, the affected population, considering the socioeconomic and educational inequity caused within the departments^(30, 31, 32)^.

Faced with the compromised psychological well-being of trans people, especially in Brazil, attention by public authorities regarding the advancement of health programs for the trans/transvestite population, such as the Transsexualization Process in the SUS^(33)^, revised by Ordinance 2.803/2013. The review of clinical/assistance protocols is still permeated by pathologizing contours, such as mandatory determination of psychological and psychiatric follow-up to obtain the medical report to start hormone therapy and endocrinologist medical follow-up^(32)^. This fact raises a necessary public debate about genocide in the Brazilian transgender population^(9)^, in the face of complex situations such as: health system bureaucratization; hormone therapy and clandestine surgeries; maintenance of illegal networks for buying/selling testosterone without a medical prescription; medical and health quackery; detachment/reluctance to gynecological care^(34)^; home insemination; stigmatization^(35)^; abandonment of psychosocial follow-up; and mental illness (anxiety, mood and personality disorders, manic episodes, depression)^(36)^.

Finally, it is important to emphasize that gender identity is one of the aspects of personality and integrates a person’s social construction, lacking a better understanding so that transgenderism is neither pathologized nor stigmatized^(37)^. In this regard, the professional category of nursing and other areas of health are called upon to dedicate attention to this problem, establishing strategies to strengthen training^(38)^ and the configuration of a new epistemology of care in nursing and health^(39)^.

### Study limitations

The limitations of this study lie in using interviews as the only resource to access narratives about the experience, which may have increased participants’ censorship in dealing with issues face to face with the researchers, in addition to not allowing a methodological triangulation and the look on the different prisms of experience. In addition, the environmentalization and acculturation preliminary to data collection were not carried out, which may have impacted the building of bonds with the sample groups and limited the scope of depth of self-reported data.

### Contributions to nursing

The study contributes to teaching and training in nursing, as it explains essential aspects for the care and assistance provided to transgender people. It also contributes to research, by evidencing new knowledge, and to practice, by indicating needs for clinical-assistance improvement and for the field of management/nursing management. Furthermore, it provides subsidies for the political emancipation of trans men and transmasculine people, encouraging the advancement of specific public policies in health systems.

## FINAL CONSIDERATIONS

Transphobia compromises the inter and intrapersonal relationships of trans men and transmasculine people, with repercussions that affect their lives and, consequently, their health, in the physical, emotional, affective and social dimensions, leaving them in a situation of high vulnerability. Data richness and saturation confirm the repercussions of transphobia and point to the need to disclose and discuss aspects that interfere with health linked to gender diversity, in order to broaden the vision about specificities about transgender people, aiming, above all, to meet the constitutional principles and the SUS and mobilize nursing care for emancipation with the intervention of different sources of transphobia, especially institutionalized in the health system.

The findings present opportunities for the field of nursing and other areas of health to act in the face of the repercussions of transphobia in the face of trans men’s and transmasculine people’s suffering. Moreover, it provided subsidies to be explored, at interdisciplinary, interprofessional and intersectoral levels, to the problem of transphobia, which goes beyond the presence of the investigated public in the environments of health services, reaching other spaces essential to human life in collectivity and, therefore, need to be taken into account from a public health dimension.
